# RANKL/RANK promotes the migration of gastric cancer cells by interacting with EGFR

**DOI:** 10.1186/s40169-019-0249-2

**Published:** 2020-01-13

**Authors:** Xing Wan, Yongxi Song, Honghong Fang, Ling Xu, Xiaofang Che, Shuo Wang, Xiaomeng Zhang, Lingyun Zhang, Ce Li, Yibo Fan, Kezuo Hou, Zhi Li, Xueqing Wang, Yunpeng Liu, Xiujuan Qu

**Affiliations:** 1 Department of Medical Oncology, The First Hospital of China Medical University, Shenyang, 110001 China; 2 Department of Surgical Oncology, The First Hospital of China Medical University, Shenyang, 110001 China; 3 Jining No.1 People’s Hospital, Shandong, 272011 China; 4 School of Medical and Health Sciences, Edith Cowan University, Perth, Australia

**Keywords:** Gastric cancer, Metastasis, Cancer biology, EGFR, RANKL, Drug targets, Translational medical research

## Abstract

**Background:**

The incidence and mortality rates of gastric cancer (GC) rank in top five among all malignant tumors. Chemokines and their receptor-signaling pathways reportedly play key roles in the metastasis of malignant tumor cells. Receptor activator of nuclear factor κB ligand (RANKL) is a member of the tumor necrosis factor family, with strong chemokine-like effects. Some studies have pointed out that the RANKL/RANK pathway is vital for the metastasis of cancer cells, but the specific mechanisms in GC remain poorly understood.

**Results:**

This study reports original findings in cell culture models and in patients with GC. Flow cytometry and western blotting analyses showed that RANK was expressed in BGC-823 and SGC-7901 cells in particular. Chemotaxis experiments and wound healing assay suggested that RANKL spurred the migration of GC cells. This effect was offset by osteoprotegerin (OPG), a decoy receptor for RANKL. RANKL contributed to the activation of human epidermal growth factor receptor (HER) family pathways. The lipid raft core protein, caveolin 1 (Cav-1), interacted with both RANK and human epidermal growth factor receptor-1(EGFR). Knockdown of Cav-1 blocked the activation of EGFR and cell migration induced by RANKL. Moreover, RANK-positive GC patients who displayed higher levels of EGFR expression had poor overall survival.

**Conclusions:**

In summary, we confirmed that with the promotion of RANKL, RANK and EGFR can form complexes with the lipid raft core protein Cav-1, which together promote GC cell migration. The formation of the RANK-Cav-1-EGFR complex provides a novel mechanism for the metastasis of GC. These observations warrant confirmation in independent studies, in vitro and in vivo. They also inform future drug target discovery research and innovation in the treatment of GC progression.

## Background

A recent study reported that the incidence and mortality rates of GC rank second among all malignant tumors [1]. The majority of patients miss the opportunity to be cured by surgery. Less than 10% of them can live for more than five years and the median survival time is less than 1 year [2]. One of the major causes of the low survival rate is local infiltration and abdominal implant transfer due to GC cell migration.

In recent years, chemokines and their receptor-signaling pathways have been reported to have major roles in the metastasis of cancer cells [3]. RANKL is the ligand of RANK, with a strong chemokine-like effect [4]. The presence of RANKL/RANK pathway has been confirmed in malignant tumors of the respiratory, endocrine, reproductive, and lymphatic systems [5–7]. The RANK/RANKL pathway has also been demonstrated to be an important regulator of mammary stem cells and mammary gland development [8]. In addition, it is vital for the initiation, progression, and metastasis of breast cancer [9, 10]. Recently, GC sufferers with high levels of RANKL expression have been confirmed to have shorter overall survival [11].

The biological function, mechanism, and significance of the epidermal growth factor receptor family have been extensively reported. EGFR and HER2 are generally considered to be markers of poor prognosis in GC sufferers [12, 13]. Studies have shown that RANK can interact with EGFR to regulate osteoclast differentiation and survival [14]. Furthermore, the interaction between EGFR and RANK has been corroborated in vitro and in animal models of metastasis. In addition, the RANK/RANKL axis also has a crucial impact on the occurrence of ErbB2-positive breast cancer [15]. Rodent experiments have shown that RANK can lead to breast cancer progression and can induce early-stage tumor metastasis in HER2-positive breast cancer models, by direct ligand stimulation [16, 17]. According to our present research, RANKL not only stimulated the migration of GC cells, but also promoted EGFR activation, which could be blocked by Cav-1 knockdown. The discovery of this RANK-Cav-1-EGFR complex revealed a novel mechanism for the metastasis of GC.

## Results

### RANKL induced the migration of GC cells via RANK and EGFR

**Fig. 1:**
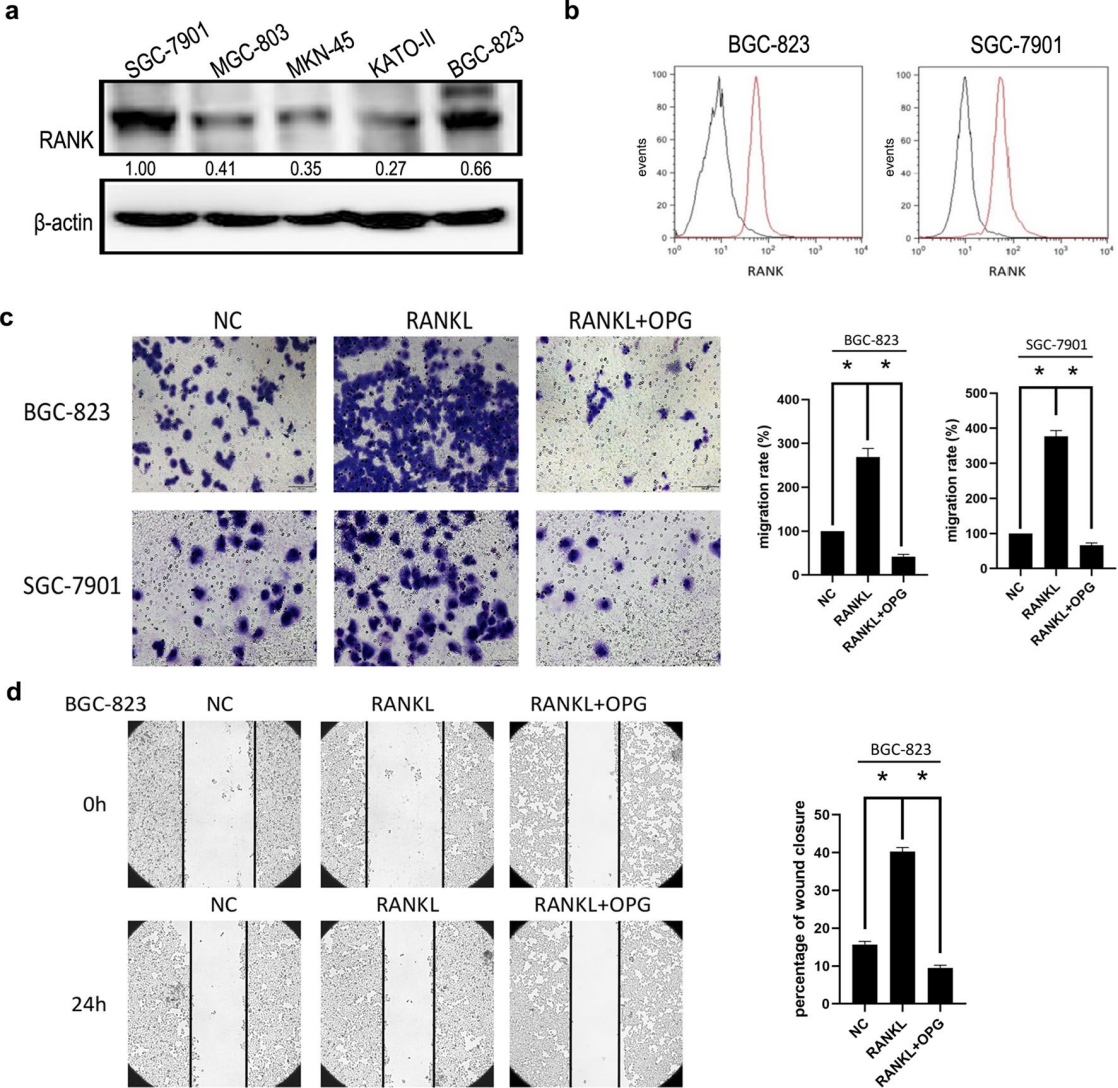
RANKL promotes gastric cancer cell migration through RANK. **a** Western blot showed that RANK protein was expressed in various gastric cancer cells. **b** The results of flow cytometry showed that there were two peaks of blue and red in gastric cancer cells, indicating that there was a high expression of RANK on the surface of gastric cancer cells. **c** SGC-7901 and BGC-823 cells were treated with RANKL (1 μg/ml) for 24 h. The transwell assay results showed that the number of cell migration increased significantly. When OPG (10 μg/ml) was added at the same time, the effect of RANKL was cancelled out. **d** The wound healing assay (magnification, × 100) also confirmed that RANKL can promote gastric cancer migration. Data are mean ± SD in three independent experiment. (**p < 0.01)

We first verified the RANK expression in various GC cells by western blot (Fig. 1a). After comparison, RANK protein expression levels were highest in BGC-823 and SGC-7901 GC cells. We then tested whether RANK was expressed on the surface of these GC cells. Flow cytometry results showed RANK expression on the surface of BGC-823 and SGC-7901 GC cells (Fig. 1b). Transwell experiments were then used to determine whether RANKL could promote GC cell migration. The results indicated that RANKL (1.0 μg/mL) promoted the migration of GC cells, compared to untreated cells. After the addition of its inhibitor, OPG (10 μg/mL), RANKL-induced GC cell migration was significantly attenuated (Fig. 1c). After being treated with the same reagent and concentration as transwell, the wound healing experiments further confirm the phenomenon above (Fig. 1d). These results suggested that RANKL stimulated the migration of GC cells through RANK.

**Fig. 2:**
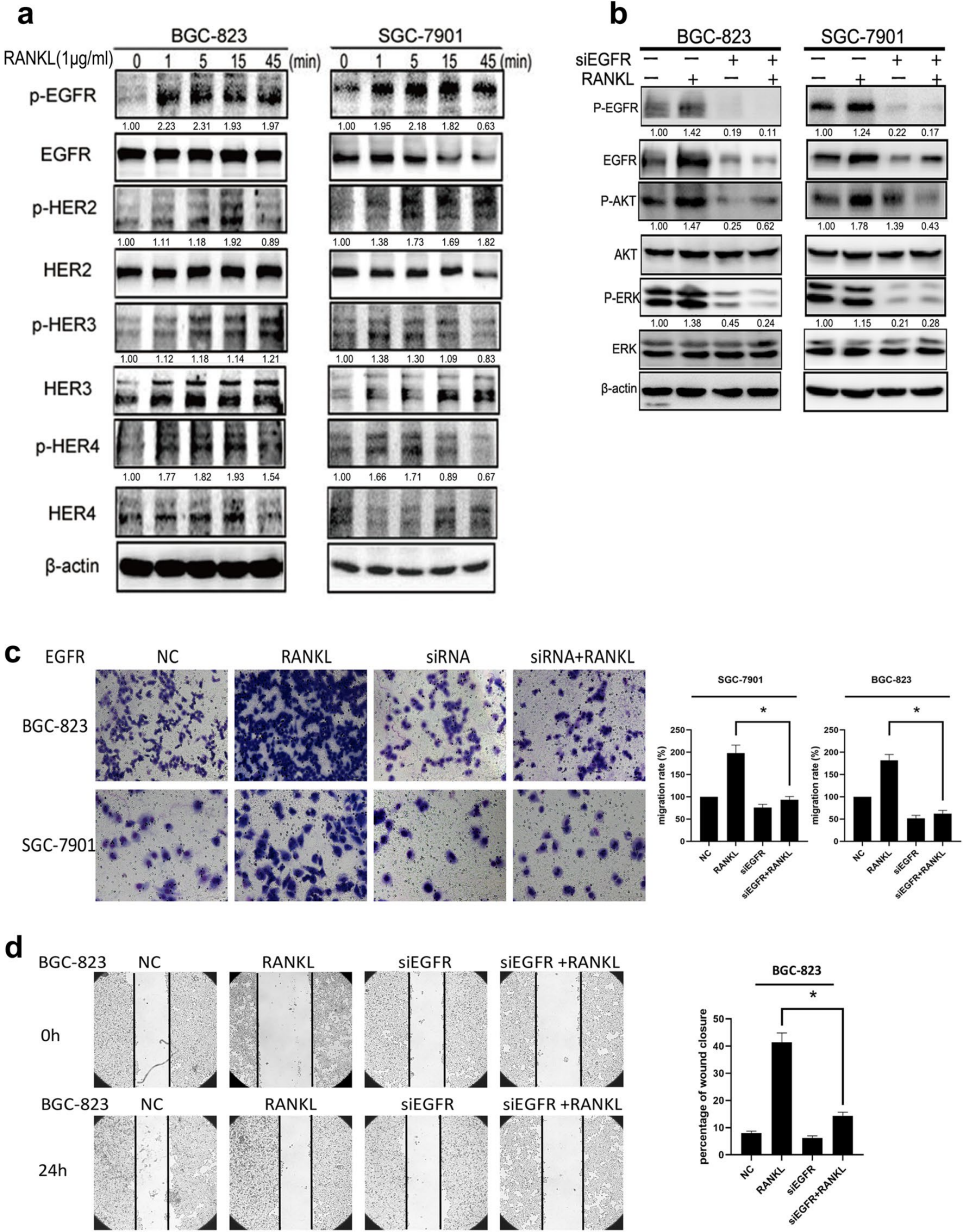
RANKL promotes migration of gastric cancer cells by activating EGFR pathway. **a** After treatment with RANKL at different time points, the HER family members showed persistent or transient phosphorylation activation by western blot. **b** RANKL can up-regulate the levels of P-EGFR, P-AKT and P-ERK. However, this effect can be counteracted by transfection of siEGFR. **c, d** The transwell assay and wound healing assay results showed that the RANKL can up-regulate the number of cell migration increased significantly. Same as above, this effect can be counteracted by transfection of siEGFR. Data are mean ± SD in three independent experiment. (**p < 0.01)

To investigate whether RANKL had an effect on the HER family, we treated both GC cells with RANKL for the indicated times and observed the level of phosphorylated EGFR. EGFR phosphorylation was obviously up-regulated at 5 min in BGC-823 and 15 min in SGC-7901. Other members of the HER family also showed transient or sustained phosphorylation (Fig. 2a). Moreover, the downstream molecular changes induced by the RANKL-mediated activation of EGFR were offset by transfection of siEGFR (Fig. 2b). Silencing EGFR also partially reversed RANKL-induced GC cell migration (Fig. 2c, d). This indicated that RANKL promoted the migration of GC cells through EGFR and its downstream signaling pathway.

### The activation of EGFR by RANKL depended on Cav-1

**Fig. 3:**
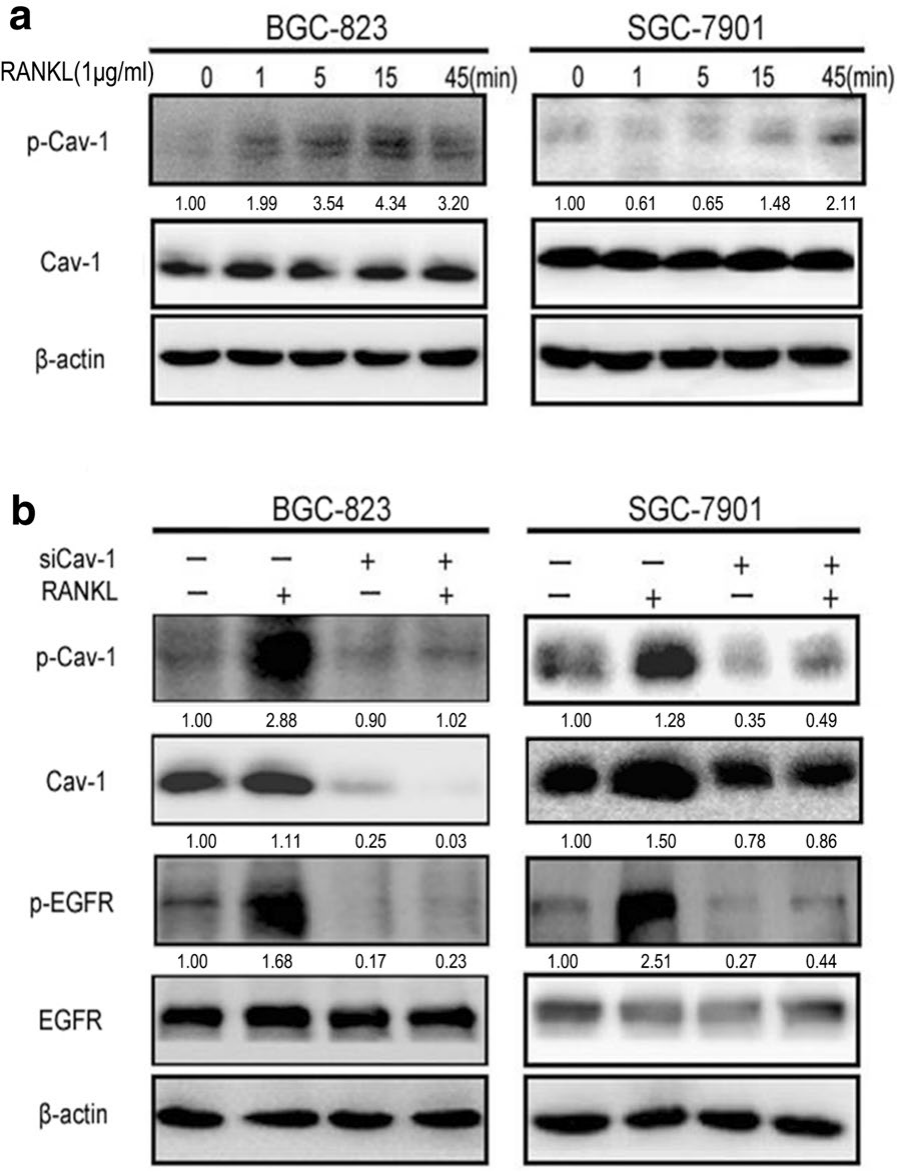
The activation of EGFR by RANKL depends on the existence of Cav-1. **a** The gastric cancer cells were treated with RANKL (1 μg/ml) for the indicated times by Western blot, the level of p-Cav-1 increased significantly, BGC-823 for 15 min and SGC-7901 for 45 min. **b** While we knocked down of Cav-1 gene by using Cav-1 siRNAs for 72 h, Cav-1 and P-Cav-1 decreased significantly, P-EGFR also decreased significantly

Numerous membrane receptors interact with each other on the lipid raft platform. To determine the mechanism whereby RANKL regulates EGFR, we examined Cav-1, a key protein of lipid rafts. The results showed that phosphorylated Cav-1 was activated by RANKL in SGC-7901 cells after 45 min of treatment and in BGC-823 cells after 15 min of treatment (Fig. 3a). Silencing of the *Cav*-*1* gene inhibited RANKL-induced EGFR activation (Fig. 3b). This result indicated that RANKL might induce GC cell migration by Cav-1-mediated EGFR activation.

### RANKL promoted GC cell migration through the formation of a RANK-Cav-1-EGFR complex

**Fig. 4:**
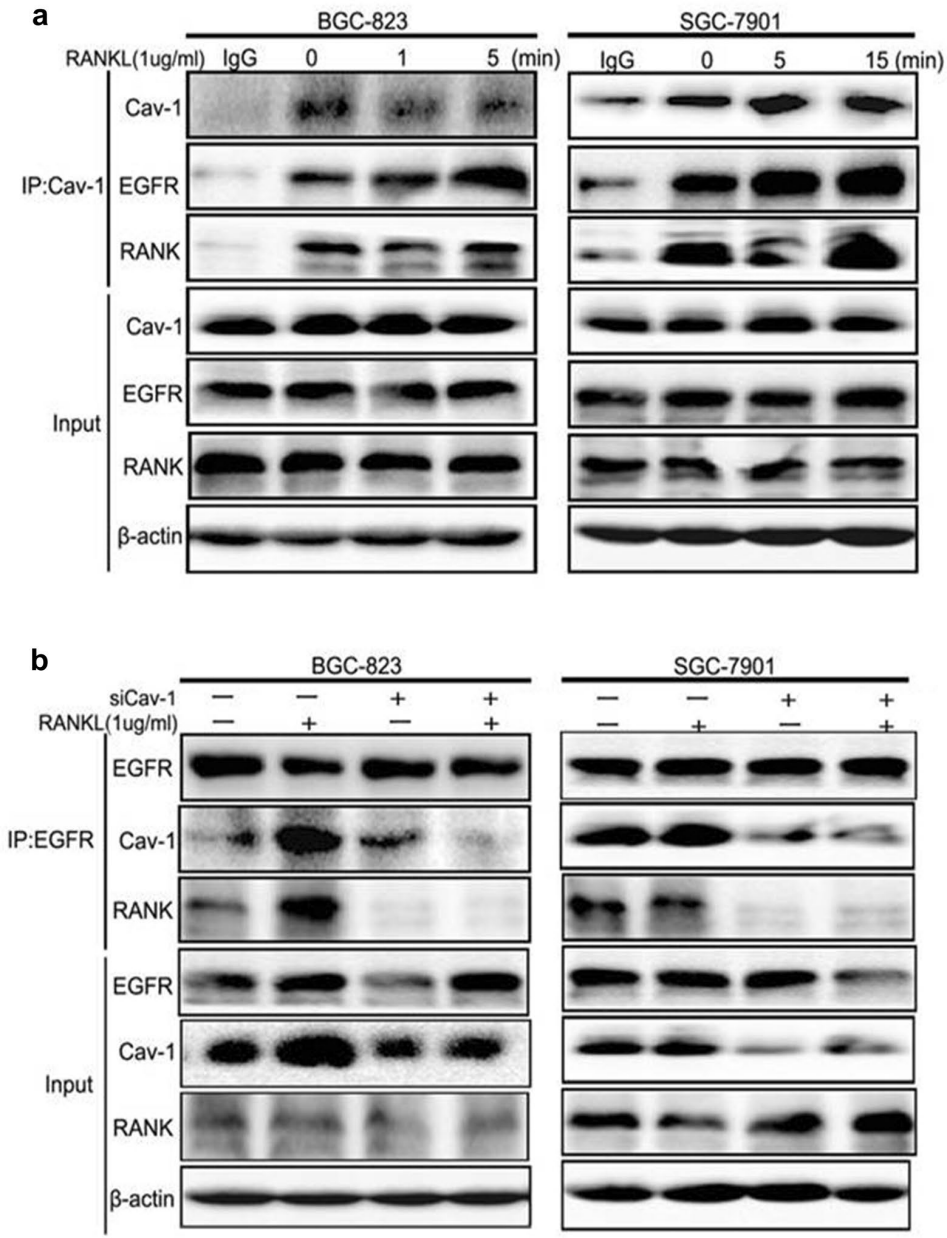
RANKL promoted the formation of a RANK-Cav-1-EGFR complex. **a** The SGC-7901 and BGC-823 cells were treated with RANKL for the indicated times. Whole cell lysates were immune-precipitated with anti-Cav-1 antibody. The interaction of CAV-1 with RANK and EGFR was significantly enhanced providing by Western blot. **b** While silencing Cav-1 gene by using Cav-1 siRNAs for 72 h, and then treated with RANKL for indicated time. The formation ability of Cav-1-RANK-EGFR complex decreased significantly. Input represents cell lysates that were not subjected to immune-precipitation and IgG as an IP-control

Since RANKL activated EGFR and Cav-1 and Cav-1 regulated EGFR activation, we explored the interaction between these proteins. Our results showed that Cav-1 naturally bound to RANK and EGFR. When treated with RANKL, the interaction of Cav-1, RANK, and EGFR increased after 5 min in BGC-823 cells and after 15 min in SGC-7901 cells (Fig. 4a). Knockdown of Cav-1 inhibited the RANK-Cav-1-EGFR complex assembling (Fig. 4b). Taken together, these findings indicated that RANKL induced GC cell migration through the formation of a RANK-Cav-1-EGFR complex.

### High levels of EGFR expression were associated with worse overall survival in RANK-positive GC sufferers

**Fig. 5:**
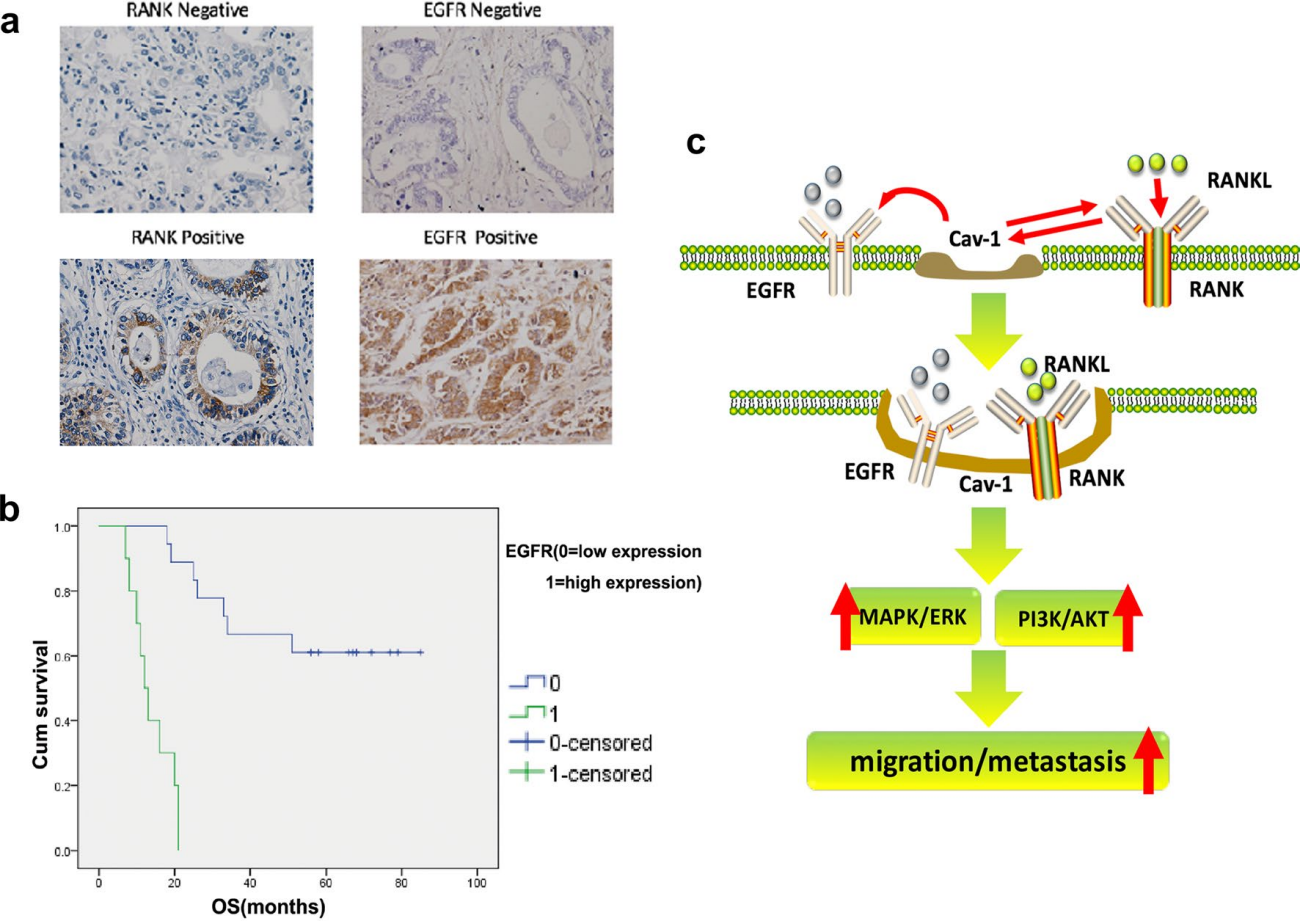
The relationship between the expression of EGFR and RANK and prognosis. **a** The cases of simultaneous negative and positive expression of EGFR and RANK. **b** The patients with double positive EGFR and RANK had the worst prognosis. **c** Schematic diagram of RANKL-mediated complex formation leading to enhanced migration of GC cells

To clarify the impact of RANK and EGFR on disease prognosis, we collected 68 primary GC specimens and used immunohistochemistry to assess EGFR and RANK expression. Immuno-staining confirmed high levels of EGFR expression in 19 patients (27.9%) and high levels of RANK expression in 28 patients (41.2%, Fig. 5a). We grouped RANK-positive patients based on their level of EGFR expression.

Table 1 shows the correlation between EGFR expression and clinic-pathological features in RANK high expression group. The univariate analysis showed that gender and age were not associated with EGFR expression. There existed a positive correlation between EGFR expression and TNM staging and N staging. We get the conclusion that the prognosis was significantly better in those with low EGFR expression sufferers than in those with high EGFR expression sufferers (Fig. 5b). This schematic diagram shows our research content very intuitively: After being stimulated by RANKL, the transmembrane proteins EGFR and RANK form a complex with Cav-1 on the lipid raft platform, which further activates downstream signaling pathways and ultimately promotes gastric cancer migration (Fig. 5c).

**Table 1:** RANK positive expression and clinicalpathological characteristics of GC

Characteristics	Cases	EGFR		
		Low (%)	High (%)	*p* value
Sex				
	Male	15 (53.6)	9 (32.1)	0.495
	Female	3 (10.7)	1 (3.6)	
Age (years)				
	≤ 60	11 (39.3)	5 (17.9)	0.590
	> 60	7 (25)	5 (17.9)	
pTNM stage				
	I + II	7 (25)	0 (0)	0.022*
	III	11 (39.3)	10 (35.7)	
T stage				
	T1-2	2 (7.1)	0 (0)	0.29
	T3-4	16 (57.1)	10 (35.7)	
N stage				
	N0	0 (0)	0 (0)	< 0.05*
	N1-3	18 (64.3)	10 (35.7)	

## Discussion

Numerous evidence supports RANK’s important function in the development and progression of cancer. RANKL stimulates RANK and has effects on the migration and invasion of cancer cells. Furthermore, RANKL/RANK function as predictors of tumorigenesis and prognosis in many solid tumors, including breast cancer and GC [18–20]. RANKL and its receptor, RANK, can be modulated by EGFR signaling [21]. However, the precise molecular mechanisms by which these two pathways interact with each other to affect cancer cell migration remain unclear.

In the present study, flow cytometry and immunoblotting experiments demonstrated that RANK exists in all five GC cell lines, with the highest levels observed in SGC-7901 and BGC-823 cells. Consistent with previous findings, RANK was located on the cell membrane. Furthermore, we examined the effect of RANKL on cell migration and verified that RANKL promoted the invading ability of GC cells. Meanwhile, the stimulation of cell migration by RANKL could be inhibited by EGFR siRNA. More importantly, by activating the EGFR signaling pathway, RANKL notably activated p-EGFR and the expression of its downstream protein, MAPK. Our data not only showed that RANK promoted tumor cell invasion through RANKL, but also suggested that there was crosstalk between the RANK/RANKL pathway and the EGFR signaling pathway.

Another important result in this study was that RANKL facilitated the phosphorylation of Cav-1, a key component of lipid rafts, which regulate signal transduction at the cell surface [22]. To further explore the molecular mechanisms of the interaction between RANKL and EGFR in the regulation of GC invasion and progression, we silenced Cav-1 protein function using a Cav-1 siRNA. The results of these experiments demonstrated that EGFR activity was regulated by RANKL, in a Cav-1-dependent manner. In particular, co-immunoprecipitation experiments revealed that Cav-1 naturally bound with RANK and EGFR. Based on the present findings, we conclude that Cav-1 may make a major contribution to the relationship between RANKL/RANK and the EGFR pathway, which are involved in regulating the migration of GC cells. These data also provide new ideas for explaining and avoiding resistance to EGFR tyrosine kinase inhibitors.

While our study suggested new evidence regarding the mechanisms of GC progression, several limitations should be noted. The clinical study sample size was limited and therefore, future studies with larger sample sizes and in independent populations are required to confirm the findings presented here. To further confirm that Cav-1 directly or indirectly interacts with RANK or EGFR and to identify the functional domain of the binding site, genetic variants of RANK, EGFR, and Cav-1 should be employed. In addition, GST pull-down experiments could be used to advance this emerging body of knowledge on GC cell migration and metastasis.

## Conclusions

The formation of RANK-Cav-1-EGFR complex provides a novel mechanism for the metastasis of GC. These observations warrant further replication in independent studies in vitro and in vivo. They also inform future drug target discovery,immunity therapy and diagnostics innovation for GC progression in particular [23].

## Materials and methods

### Clinical study, participants, and research ethics

Samples of GC tissue were gathered from 68 sufferers who received Radical surgery at the First Hospital of China Medical University (Shenyang, China) from March 2006 to October 2011. No one had ever received any form of anti-tumor treatment. Clinical and pathological features were evaluated following medical charts and pathological records. The Ethical Committee of the First Hospital of China Medical University approved the study.

### Reagents

RANKL and OPG were obtained from CytoLab/PeproTech Asia (Rocky Hill, NJ, USA). The Cav-1 siRNA was obtained from Shanghai GenePharma Co. Ltd (Shanghai, China). The EGFR siRNA and protein agarose beads were purchased from Cell Signaling Technology, Inc. (Danvers, MA, USA). Lipofectamine 2000 was purchased from Invitrogen (Carlsbad, CA, USA).

### Cell culture

Five GC cells were purchased from the Cell Bank of the Chinese Academy of Sciences (Shanghai, China). The specific methods refer to our previous article [24].

### Flow cytometry

BGC-823 and SGC-7901 cells were sowed at 2.5 × 10^5^ cells/well in six-well plates. RANK expression was detected by flow cytometry according to a previously described method [25].

### Western blotting

Western blotting was performed as previously described [25, 26].

### Immunoprecipitation assay

Antibodies against EGFR and Cav-1, protein A-agarose beads, and cell lysates were incubated for 6 h at room temperature. The resulting complex was washed four times with lysis buffer. The technical details of this procedure are described in our previous publication [27].

### Transwell migration assay

For each sample, 200 μl of pretreated cells in serum-free RPMI 1640 medium (SGC-7901, 1 × 10^4^ cells/well; BGC-823, 3 × 10^4^ cells/well; *EGFR* or *Cav*-*1* gene knocked out) was loaded into the upper well. The lower chambers supplemented with or without RANKL. The specific experimental methods refer to our previous article [24].

### Wound healing assay

For detailed experimental methods, please refer to our previous research [28].

### Transfection with small interfering RNA

The cultured cells were transiently transfected by using Lipo 2000 at the request of the instructions. The following siRNA sequences were used: siCav-1, 5′-AACCAGAAGGGACACACAGUU-3′; EGFR, 5′-CUCCAGAGGAUGUUCAAUATT-3′; and control, 5′-AATTCTCCGAACGTGTCACGT-3′. After 48–72 h of transfection, the cells were sub-cultured for further experiments.

### Immunohistochemical experiments

GC tissues were formalin-fixed, paraffin-embedded, and cut into 3 µm sections. The specific details of the immune-histochemical method have been described in our previous report [27]. Briefly, the following antibodies were used for immune-histochemical staining: an anti-RANK antibody from R&D Systems (Minneapolis, MN, USA) and an anti-EGFR antibody from Santa Cruz Biotechnology, Inc. (Dallas, TX, USA). Two independent pathologists observed and evaluated the stained tissue sections by microscopy ( ×  20 and  ×  40 magnification). Five fields were randomly selected from each section and scored based on the percentage of positive cells and the staining intensity of the cells. Sections with 0–10, 10–25, 26–50, 51–75, or > 76% of positively stained cells were recorded as 0, 1, 2, 3, and 4, respectively. Scores > 2 were defined as high expression levels and scores of 0–2 were defined as low expression levels.

### Statistical analysis

All experiments were performed on at least three independent occasions. Data were summarized and are showed as the mean ± SD. Statistical comparisons were performed using a Student’s t-test, with *p* < 0.05 considered statistically significant. Statistical analysis was performed using SPSS 24.0 software (SPSS, Inc., Chicago, IL, USA).

## Data Availability

Data sharing is not applicable to this article as no datasets were generated or analyzed during the current study.

## Abbreviations

Akt: serine/threonine kinase
Cav-1: caveolin 1
EGFR: human epidermal growth factor receptor-1
ERK: extracellular regulated kinase
FBS: fetal calf serum
GC: gastric cancer
HER2: human epidermal growth factor receptor-2
HER3: human epidermal growth factor receptor-3
HER4: human epidermal growth factor receptor-4
MAPK: mitogen-activated protein kinase
OPG: osteoprotegerin
P-EGFR: phosphorylation human epidermal growth factor receptor-1
P-HER2: phosphorylation human epidermal growth factor receptor-2
P-HER3: phosphorylation human epidermal growth factor receptor-3
P-HER4: phosphorylation human epidermal growth factor receptor-4
PI3K: phosphatidylinositol 3-kinase
PMSF: phenylmethylsulfon
RANK: receptor activator of nuclear factor-κ B
RANKL: receptor activator of nuclear factor-κ B ligand
